# Meta-analysis of adverse health effects due to air pollution in Chinese populations

**DOI:** 10.1186/1471-2458-13-360

**Published:** 2013-04-18

**Authors:** Hak-Kan Lai, Hilda Tsang, Chit-Ming Wong

**Affiliations:** 1 Department of Community Medicine, School of Public Health, The University of Hong Kong, Hong Kong, SAR, China

**Keywords:** Air pollution, Health and mortality, Systematic review, Relative risk, Random effects, Fixed effect

## Abstract

**Background:**

Pooled estimates of air pollution health effects are important drivers of environmental risk communications and political willingness. In China, there is a lack of review studies to provide such estimates for health impact assessments.

**Methods:**

We systematically searched the MEDLINE database using keywords of 80 major Chinese cities in Mainland China, Hong Kong and Taiwan on 30 June 2012, yielding 350 abstracts with 48 non-duplicated reports either in English or Chinese after screening. We pooled the relative risks (RR) per 10 μg/m^3^ of particulate matter (PM_10_), nitrogen dioxide (NO_2_), sulphur dioxide (SO_2_) and ozone (O_3_).

**Results:**

For short-term effects, the pooled RR (p < 0.05) ranges were: 1.0031 (PM_10_) to 1.0140 (NO_2_) for all-cause mortality, 1.0034 (cardiopulmonary, PM_10_) to 1.0235 (influenza and pneumonia, SO_2_) for 9 specific-causes mortality, 1.0021 (cardiovascular, PM_10_) to 1.0162 (asthma, O_3_) for 5 specific-causes hospital admissions. For birth outcomes, the RR (p < 0.05) ranged from 1.0051 (stillbirth, O_3_) to 1.1189 (preterm-birth, SO_2_) and for long-term effect on mortality from 1.0150 (respiratory, SO_2_) to 1.0297 (respiratory, NO_2_). Publication bias was absent (Egger test: p = 0.326 to 0.624). Annual PM_10_ and NO_2_ concentrations were inversely associated with RR of mortality (p = 0.017-0.028).

**Conclusions:**

Evidence on short-term effects of air pollution is consistent and sufficient for health impact assessment but that on long-term effects is still insufficient.

## Background

Air pollution has been a worldwide problem in both outdoor and indoor environment. Among all air pollutants, the most commonly monitored are particulate matter (PM), nitrogen dioxide (NO_2_), sulphur dioxide (SO_2_) and ozone (O_3_). The World Health Organization (WHO) had reviewed the health effects of these four pollutants and recommended them in the Air Quality Guidelines early in 1987 [1] and the latest in 2005 [2].

Epidemiologic evidence which showed the link between the ambient level of air pollution and adverse respiratory and cardiovascular outcomes [3,4] was mainly based on assessment of excess mortality numbers associated with short-term exposures. Examples include the APHEA and NMMAPS studies in both Europe and the US [5-7]. Meta-analysis and review studies have been conducted to pool the short-term health effects of air pollutants in many countries [8-10] but seldom in China. The concentration-response gradient for air pollutants from other countries may not be applicable to Chinese populations due to the differences in community settings, pollutant compositions, time-microenvironment-activity patterns, culture, lifestyles, genetic susceptibility, and the much higher exposure levels in China where saturation mechanism may reduce the exposure-response gradients [11]. The lack of pooled risk estimates due to air pollution in China would limit health impact assessments that are important drivers of environmental risk communications and political willingness. A successful application of the pooled risk estimates in the present study has been demonstrated in another paper on the health impact assessment of marine emissions in Pearl River Delta [12], supporting environmental policy decisions in a timely manner.

With the rapid economic growth in China, the level of air pollution from both motor vehicles and industrial emissions has drastically increased. Compared to other countries, such as the United States and the United Kingdom, potential health effects of increasing air pollution in China remain largely unmeasured, with the exception of results from the PAPA study in Asia [13], Kan’s study from four reports in China [14], and Zhou’s study in Yangtze River Delta [15]. At present, there were three studies found in PubMed (http://www.ncbi.nlm.nih.gov/pubmed) about meta-analysis of adverse effects of air pollutants in Chinese population [14,16,17] with pooled risk estimates for PM_10_ and SO_2_ in mainland China and Hong Kong only but not for NO_2_ and O_3_.

In this study, a systematic review and a meta-analysis were carried out to pool the risk estimates for mortality and morbidity outcomes due to the four classical air pollutants [2], PM_10_, NO_2_, SO_2_ and O_3_, in the Chinese populations including Mainland China, Hong Kong and Taiwan.

## Methods

We searched the MEDLINE database (last entry on 30th June 2012) using the following terms for titles and abstracts: (“particulate matter” OR “PM10” OR “PM(10)” OR “nitrogen dioxide” OR “NO2” OR “NO(2)” OR “sulfur dioxide” OR “sulphur dioxide” OR “SO2” OR “SO(2)” OR “ozone” OR “O3” OR “O(3)”) AND health AND (“China” OR “Taiwan” OR “river delta” OR “Chinese”). We also added terms for the names of 80 major Chinese locations (with population size ranged from 0.5 to 29 millions. See Additional file 1), yielding 26 locations with relevant literatures including Anshan, Beijing, Chongqing, Foshan, Fuzhou, Guangzhou, Hangzhou, Hong Kong, Kaohsiung, Lanzhou, Nanjing, Quanzhou, Shanghai, Shenyang, Shenzhen, Suzhou, Taichung, Taipei, Taiyuan, Tangshan, Tianjin, Urumqi, Wuhan, Xian, Zhongshan and Zhuhai.

**Figure 1:**
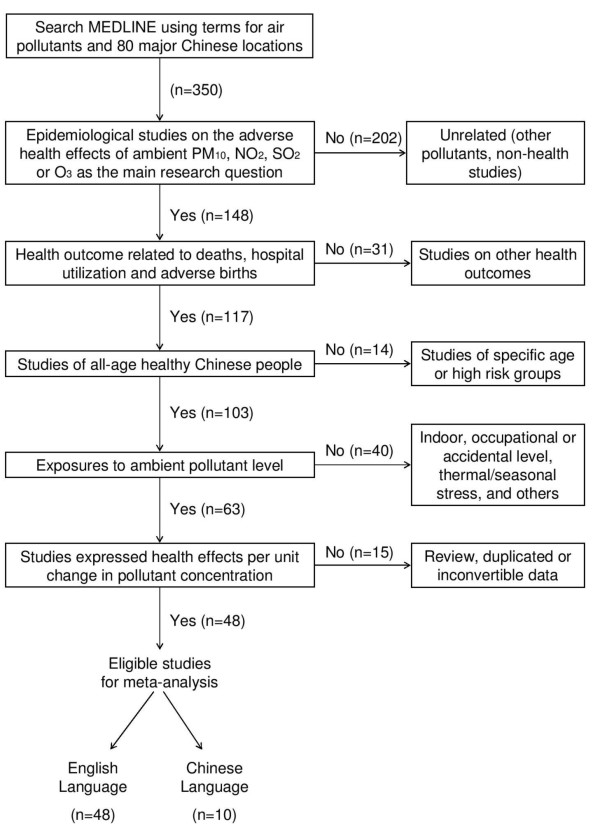
Systematic screening stage for literature review.

There were 350 abstracts retrieved from PubMed on 30th June 2012 and 48 literatures were selected for this review by a researcher using the following inclusion criteria: (i) All epidemiologic studies on the adverse health effects of PM_10_, NO_2_, SO_2_ or O_3_ in Chinese population as the main research question; (ii) the health outcomes were related to deaths, births and hospital utilization; (iii) the subjects were not designed to specific high risk groups (e.g. patients or smokers) nor specific age subgroups (e.g. children or elders); (iv) exposures to ambient levels (not indoor, occupational or accidental exposures); (v) the health risk estimates were expressed in terms of unit change in pollutant mass concentration; and (vi) reported in English or Chinese. Duplicate publication of the same results would be included only once (Figure 1). The study was adhered to the PRISMA guidelines [18].

### Meta-analysis

Meta-analysis [19,20] for each adverse health outcome reported by two or more studies was conducted to estimate the pooled estimates effects of PM_10_, NO_2_, SO_2_ and O_3_ on mortality (all-natural and cause-specific mortality), hospital admissions and emergency room visits. Quantities of risk estimates from the selected studies were summarized using the overall average of these quantities weighted by the inverse variance. All risk estimates were expressed as or converted to percentage change in the number of adverse health events associated with every 10 μgm^-3^ change in pollutant concentration. Only the single-pollutant model results were included. When there were more than one publications on the same population, the one from the latest study was selected in order to avoid over-representation (Figure 1). For short-term risk estimates, lags ranged within day 0 and 1 were used since they were mostly reported [13,21]. We first included risk estimates with the single lag day (either 0 or 1) that is more significantly larger than unity with a smaller p-value, then included estimates of an average lag day (0–1) only if estimates of single lag were not reported. When there was no report for lag 0, 1 or 0–1, we included lag day 2 and an average lag day 0–2. In sensitivity analysis, we included risk estimates that were only reported with lags more than two days. When pooling the estimates, we used random effects if *I*^2^ statistics for heterogeneity was >25% or otherwise the fixed effect [19].

### Heterogeneity and publication bias

The overall relative risk (RR) for mortality due to cardiovascular diseases (CD) and respiratory diseases (RD) were pooled; and diagnosis for overall heterogeneity due to each study was assessed by influence plots [19]. Funnel plots with Egger test on asymmetry at alpha level 0.1 was used for assessment of publication bias [22]. The overall heterogeneity was assumed significant under normal distribution when the square root of *q*^*2*^ statistic laid outside 95% of all statistic values to be between −1.96 and 1.96 [19].

### Sensitivity analysis

The rubrics were identified according to the diseases ICD code instead of the description of the authors. Sensitivity analysis was done to assess the differences in the usage of ICD codes for the same rubric between studies. We calculated by the percentage difference between the code ranges that were most commonly used and the code ranges that were less common (See Additional file 1). The median percentage difference among all rubrics was 40%. The risk estimates in each rubrics were pooled again using studies with percentage difference ≤40%. For studies without ICD codes reported, the authors of these papers were contacted in both Chinese and English to provide code information.

## Results

### Air pollutant concentrations

**Figure 2:**
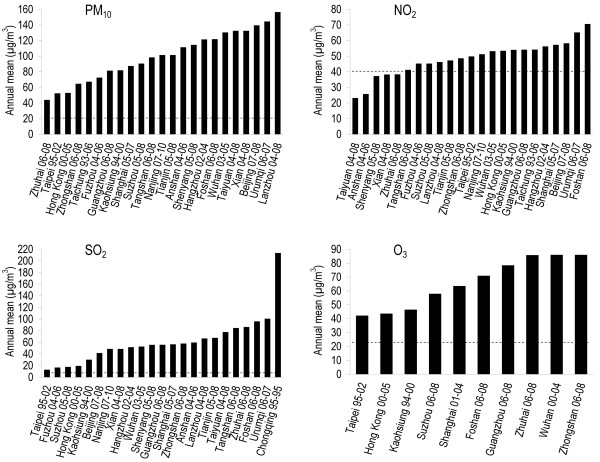
**Air pollution concentration in Chinese cities.** Only the annual mean concentrations of the latest publication for each city were shown to avoid over-representation. The years of the study period were indicated after the city names. Dotted lines for PM_10_ and NO_2_ were WHO annual Air Quality Guidelines [23]. Dotted lines for SO_2_ and O_3_ were annual limits derived from WHO short-term Air Quality Guidelines [24].

Studies from the Mainland China, Taiwan and Hong Kong reported from 1989 to 2010. The annual mean concentrations ranged 44–156 μg/m^3^ for PM_10_, 23–70 μg/m^3^ for NO_2_, 14–213 μg/m^3^ for SO_2_ and 34–86 μg/m^3^ for O_3_ (Figure 2). All relative risks (RR) [95% confidence intervals] in the following contexts were based on 10 μg/m^3^ increase in one pollutant concentration.

### Mortality

#### Short-term exposures

Estimates of the effect of exposure to daily concentration of air pollutants on daily mortality numbers were reported by 26 studies in 24 Chinese jurisdictions including Anshan, Beijing, Chongqing, Foshan, Fuzhou, Guangzhou, Hangzhou, Lanzhou, Shanghai, Shenyang, Suzhou, Taiyuan, Tangshan, Tianjin, Urumqi, Wuhan, Xian, Zhongshan, Zhuhai; Kaohsiung, Taichung, Taipei, and Hong Kong (Tables 1 and 2).

**Table 1:** Relative risks of all-cause mortality in all age groups due to air pollution in different reviewed studies

**Ref#**	**City**	**Period**	**PM**_**10**_		**NO**_**2**_		**SO**_**2**_		**O**_**3**_	
			**RR**	**SE**	**RR**	**SE**	**RR**	**SE**	**RR**	**SE**
[25]	Anshan	2004–2006	1.0024	14	1.0130	70	1.0027	17		
[26,27]	Beijing	2007–2008	1.0017*	5	1.0040	24				
[28]		1989					1.0107*	25		
[29]	Chongqing	1995					1.0030	26		
[30]	Foshan	2006–2008	1.0050*	10	1.0187*	24			1.0036	21
[26,27]	Fuzhou	2004–2006	1.0063*	23	1.0271*	106				
[30]	Guangzhou	2006–2008	1.0074*	11	1.0166*	20			1.0064*	11
[26,27]	Hangzhou	2002–2004	1.0032*	14	1.0095*	46				
[26,27,31]	Hong Kong	1996–2002	1.0064*	12	1.0122*	13	1.0091*	26	1.0034*	16
[32]	Kaohsiung	1994–2000	1.0000	43	1.0003	87	1.0040	111	0.9984	56
[26,27]	Lanzhou	2004–2008	1.0001	5	1.0054*	22				
[26,27]	Nanjing	2007–2010			1.0107*	28				
[33]	Shanghai	2004–2005	1.0016*	7						
[26,27,34]		2001–2004			1.0127*	13	1.0095*	17	1.0031*	14
[26,27]	Shenyang	2005–2008	1.0018*	8	1.0150*	30				
[26,27]	Suzhou	2005–2008	1.0037*	11	1.0173*	27				
[35]		2006–2008							1.0036*	15
[36]	Taichung	1993–2006	1.0039*	10	1.0016	16				
[37]	Taipei	1994–1998	0.9984	41	1.0015	63	0.9982	135	0.9995	75
[26,27]	Taiyuan	2004–2008	1.0021*	9	1.0422*	70				
[26,27]	Tangshan	2006–2008	1.0011	19	1.0141*	58				
[26,27]	Tianjin	2005–2008	1.0075*	16	1.0246*	58				
[38]		2005–2007					1.0056*	17		
[26,27]	Urumqi	2006–2007	1.0010	10	1.0085	60				
[26,27]	Wuhan	2003–2005	1.0042*	8	1.0290*	25				
[39]		2000–2004					1.0120*	28	1.0029	17
[26,27]	Xian	2004–2008	1.0019*	8	1.0225*	36				
[30]	Zhongshan	2006–2008	1.0044	24	1.0122*	40			1.0061*	20
[30]	Zhuhai	2006–2008	1.0037	69	1.0139	77			1.0022	30
		**Pooled RR**^**a**^	**1.0031**^R^*	**5**	**1.0140**^R^*	**11**	**1.0071**^R^*	**13**	**1.0042**^F^*	**6**

*Note. RR*, Relative risk per 10 μg/m^3^ increase in pollutant concentration; *SE*, Standard error in 10^-4^. ^R^ represented random effects. ^F^ represented fixed effects. * indicated statistically significant at alpha = 0.05. 95% Cl = 1.96*SE ± RR.

**Table 2:** Summary of pooled relative risks of different health outcomes due to air pollution

	**PM**_**10**_		**NO**_**2**_		**SO**_**2**_		**O**_**3**_	
**Health outcome [Ref. #]**	**RR**	**SE**	**RR**	**SE**	**RR**	**SE**	**RR**	**SE**
***Mortality***								
**All causes**[25-40]	**1.0031**^R^*	5	**1.0140**^R^*	17	**1.0071**^R^*	9	**1.0042**^F^*	6
**Cardiovascular diseases**[25-37,39,41]	**1.0049**^R^*	7	**1.0162**^R^*	22	**1.0072**^R^*	17	**1.0051**^R^*	13
**Respiratory diseases**[25-27,30-33,35,37,39,40,42,43]	**1.0057**^R^*	9	**1.0220**^R^*	33	**1.0129**^R^*	36	**1.0048**^F^*	14
**Cardiopulmonary diseases**[34,39,43]	**1.0034**^F^*	6	**1.0155**^R^*	54	**1.0123**^F^*	15	**1.0023**^**F**^	15
**Cardiac diseases**[30,31,34,39]	**1.0062**^R^*	15	**1.0177**^F^*	23	**1.0182**^R^*	41	**1.0026**^**R**^	31
**Cerebrovascular diseases**[30,31,34,36,39]	**1.0057**^R^*	18	**1.0147**^R^*	39	**1.0079**^F^*	26	**1.0057**^R^*	29
**Ischemia heart diseases**[36,44]	0.9963	52	**1.0130**^**R**^	118	1.0280*	82		
**Chronic obstructive pulmonary diseases**[28-31,45]	**1.0065**^**R**^	41	**1.0184**^R^*	58	**1.0095**^R^*	43	**1.0071**^**R**^	39
**Influenza and pneumonia**[31,34]	**1.0105**^F^*	33	**1.0175**^F^*	49	**1.0235**^F^*	70	1.0054^**F**^	45
**Non-cardiopulmonary**[31,34,39]	**1.0028**^F^*	6	**1.0094**^R^*	19	**1.0076**^F^*	16	**1.0025**^F^*	10
**Cancer**[28,29,43]	1.0031*	15			**1.0020**^**R**^	14		
**Diabetes**[46]	1.0060	31	1.0130	66	1.0110	320		
**Accidental**[31,34]	**1.0015**^**F**^	22	**1.0000**^**F**^	46	**0.9988**^**F**^	56	**1.0005**^**R**^	59
***Hospital admissions***								
**All causes**[47]	0.9972	17	1.0014	49	1.0003	34		
**Cardiovascular diseases**[31,47]	**1.0021**^R^*	10	**1.0095**^F^*	21	**1.0079**^R^*	43	1.0024	11
**Respiratory diseases**[31,47]	**1.0039**^**R**^	22	**1.0060**^R^*	24	**1.0014**^**F**^	15	1.0081*	12
**Cardiac diseases**[31]	1.0058*	11	1.0100*	13	1.0098*	21	1.0012	13
**Cerebrovascular diseases**[31,48]	**0.9981**^**R**^	40	**1.0032**^**F**^	21	**0.9986**^**F**^	31	**0.9994**^**F**^	19
**Ischemia heart diseases**[31,48]	**1.0065**^F^*	20	**1.0142**^**R**^	74	**1.0102**^F^*	36	**1.0024**^**F**^	21
**Asthma**[31]	**1.0077**^F^*	24	**1.0094**^F^*	31	**0.9992**^**F**^	47	**1.0162**^F^*	30
**Chronic obstructive pulmonary diseases**[31]	1.0132*	17	1.0194*	20	1.0070*	31	1.0154*	19
**Influenza and pneumonia**[31]	1.0066*	20	1.0076*	24	1.0009	38	1.0109*	23
**Pneumonia**[48]	1.0006	47	1.0071	84	1.0062	146	1.0081	81
**Acute respiratory diseases**[31]	1.0088*	20	1.0122*	25	1.0055	38	1.0155*	22
***Emergency admissions***								
**Cardiovascular diseases**[49]	1.0060*	26	1.0130*	36	1.0160*	51		
**Respiratory diseases**[49]					1.0130*	41		
**Ischemia heart diseases**[49,50]	1.0070	41	1.0100	51	1.0100	77		
**Cerebrovascular diseases**[49]	1.0030	36	1.0080	51			0.9920	46
**Cardiac diseases**[50]	1.0050*	20	1.0120*	26	1.0160*	31	1.0045*	21
**Heart failure**[49]					1.0360*	117		
**Asthma**[51]	1.0060*	20	1.0090*	26	1.0040	36	1.0150*	20
**Chronic obstructive pulmonary diseases**[51]	1.0050*	10	1.0090*	20	1.0070	36	1.0110*	15
***Emergency room visit***								
**All causes**[52]	1.0002	5	1.0070*	14	1.0023	10		
**Cardiovascular diseases**[53,54]	1.0030	15	1.0140	71	1.0140*	51		
**Respiratory diseases**[55,56]	1.0011	23	1.0050	38	1.0010	20		
**Ischemia heart diseases**[53]	1.0010	46						
**Cerebrovascular diseases**[50]	1.0010	31						
**Heart failure**[50]	1.0120	97						
**Cardiac arrhythmias**[50]	1.0020	51						
**Hypertension**[57,58]	1.0188	69	1.0689	286	1.0370*	173		
**Acute upper respiratory infections**[55]	1.0130*	20	1.0320*	77	1.0180*	46		
**Acute pharyngitis**[55]	1.0120*	46	1.0300	168	1.0170	102		
***Adverse birth outcomes***								
**Stillbirth**[59]	1.0200	153	1.0053	163	1.0076	39	1.0051	130
**Preterm birth**[60]	1.0442*	144	1.0543*	186	1.1189*	265	1.0463*	218
**Low birth weight – 3rd trimester**[61]					1.0110*	26		
**Low birth weight – entire pregnancy**[62]					1.0087	46		

*Note.* RR, with **Bold** represents pooled relative risk per 10 μg/m^3^ increase in pollutant concentration, RR not **Bold** was the RR of single study. SE, Standard error in 10^-4^. ^R^ represented random effects. ^F^ represented fixed effects. * indicated statistically significant at alpha = 0.05. 95% Cl = 1.96*SE ± RR.

The pooled RR of all-cause mortality were 1.0031 [1.0022-1.0041] for PM_10_, 1.0140 [1.0106-1.0174] for NO_2_, 1.0071 [1.0045-1.0097] for SO_2_ and 1.0042 [1.0031-1.0053] for O_3_. All the cities reported statistically significant associations between daily mortality and all the four pollutants except PM_10_ in Lanzhou, Tangshan, Urumqi and Zhongshan, NO_2_ in Beijing, Taichung and Urumqi, SO_2_ in Chongqing and O_3_ in Fushan and Wuhan. The associations were not significant (95% CI including unity) for all pollutants in Anshan, Kaohsiung, Taipei and Zhuhai.

For cause-specific mortality, the pooled RR of CD mortality were 1.0049 [1.0034-1.0063] for PM_10_, 1.0162 [1.0118-1.0205] for NO_2_, 1.0072 [1.0039-1.0105] for SO_2_ and 1.0051 [1.0025-1.0077] for O_3_. The pooled RR of RD mortality were 1.0057 [1.0040-1.0075] for PM_10_, 1.0220 [1.0156-1.0284] for NO_2_, 1.0129 [1.0058-1.0199] for SO_2_ and 1.0048 [1.0019-1.0076] for O_3_. The pooled RR of cardiopulmonary mortality were 1.0034 [1.0023-1.0046] for PM_10_, 1.0155 [1.0049-1.0261] for NO_2_ and 1.0123 [1.0093-1.0153] for SO_2_. For other specific causes, all the four pollutants were associated with cerebrovascular mortality, whereas NO_2_ and SO_2_ were associated with mortality for COPD. PM_10_, NO_2_ and SO_2_ were associated with mortality for influenza and pneumonia, as well as cardiac diseases.

The pooled RR estimate of all the statistically significant results (p < 0.05) for all cause mortality ranged from 1.0031 (PM_10_) to 1.0140 (NO_2_), for CD from 1.0049 (PM_10_) to 1.0162 (NO_2_), and for RD from 1.0048 (O_3_) to 1.0220 (NO_2_).

#### Long-term exposures

Estimates of the effect of exposure to annual average concentration of air pollutants on mortality were reported by 3 cohort studies which covered 32 cities in mainland China. Data of RR were not yet sufficient for meta-analysis at present (Table not shown).

In one cohort studies in Shenyang, the RR of all causes of mortality were 1.0153 [1.0150-1.0156] for PM_10_ and 1.0245 [1.0234-1.0258] for NO_2_; the corresponding RR of CD mortality were 1.0155 [1.0151-1.0160] and 1.0246 [1.0231-1.0263], of cerebrovascular mortality 1.0149 [1.0145-1.0153] and 1.0244 [1.0227-1.0262] [63], and of RD mortality 1.0167 [1.0160-1.0174] and 1.0297 [1.0269-1.0327] [64], respectively. In another cohort study examining the effects of SO_2_ in 31 cities in mainland China reported that RR of all-cause, CD and RD mortality were 1.018 [1.013-1.023], 1.032 [1.023-1.040] and 1.015 [1.003-1.028] respectively [65].

### Morbidity

Exposure to daily air pollutant concentrations were associated with daily total numbers of hospital admissions, emergency room visits and emergency admissions, mainly for CD and RD, which included sub-categories cardiac diseases, ischemia heart diseases (IHD), cerebrovascular diseases, heart failure, hypertension, asthma, COPD, and influenza and pneumonia (Table 2). Associations with pre- and postnatal outcomes included stillbirths, post-neonatal deaths, preterm births and low birth weight reported by 22 studies in Beijing, Hong Kong, Shanghai, Taipei and the whole Taiwan (Table 2).

For hospital admissions, the pooled RR of CD among 2 cities were 1.0021 [1.0002-1.0040] for PM_10_ and 1.0095 [1.0054-1.0137] for NO_2_; that of RD were 1.0060 [1.0012-1.0107] for NO_2_. The pooled RR of IHD were 1.0065 [1.0027-1.0104] for PM_10_ and 1.0102 [1.0031-1.0172] for SO_2_; that of asthma were 1.0077 [1.0029-1.0125] for PM_10_, 1.0094 [1.0032-1.0155] for NO_2_ and 1.0162 [1.0103-1.0221] for O_3_. All the four pollutants were associated with COPD hospital admissions in Hong Kong. Acute respiratory diseases, influenza and pneumonia, and cardiac diseases were reported in Hong Kong only.

For emergency admissions, the associations of CD, RD, cardiac diseases, heart failure, asthma, and COPD reported in Hong Kong only. For emergency room visits, the RR of daily visits were associations in Shanghai only. For specific causes, the association of CD, hypertension, cardiac arrhythmias, acute upper respiratory infections and acute pharyngitis were reported in Beijing only (Figure 2).

For adverse birth outcomes, in Taiwan, the RR of stillbirth was 1.02 [1.00-1.05] in the first and 1.02 [1.00-1.04] in the second month of gestation for PM_10_ and 1.0076 [1.000-1.0153] in the first-trimester for SO_2_[59]. In Shanghai, sub-chronic exposure (8-week average) to the PM_10_, NO_2_, SO_2_ and O_3_ corresponded to RR of preterm births of 1.0442 [1.0160-1.0725], 1.0543 [1.0178-1.0908], 1.1189 [1.0669-1.1709] and 1.0463 [1.0035-1.0891], respectively [60]. For exposure to SO_2_ in Beijing, the RR of low birth weight was 1.011 [1.006-1.016] [61]. In Taipei, the RR of low birth weight was 1.0087 [1.0013-1.0177] for SO_2_ maternal exposure during pregnancy [62].

### Heterogeneity and publication bias

**Figure 3:**
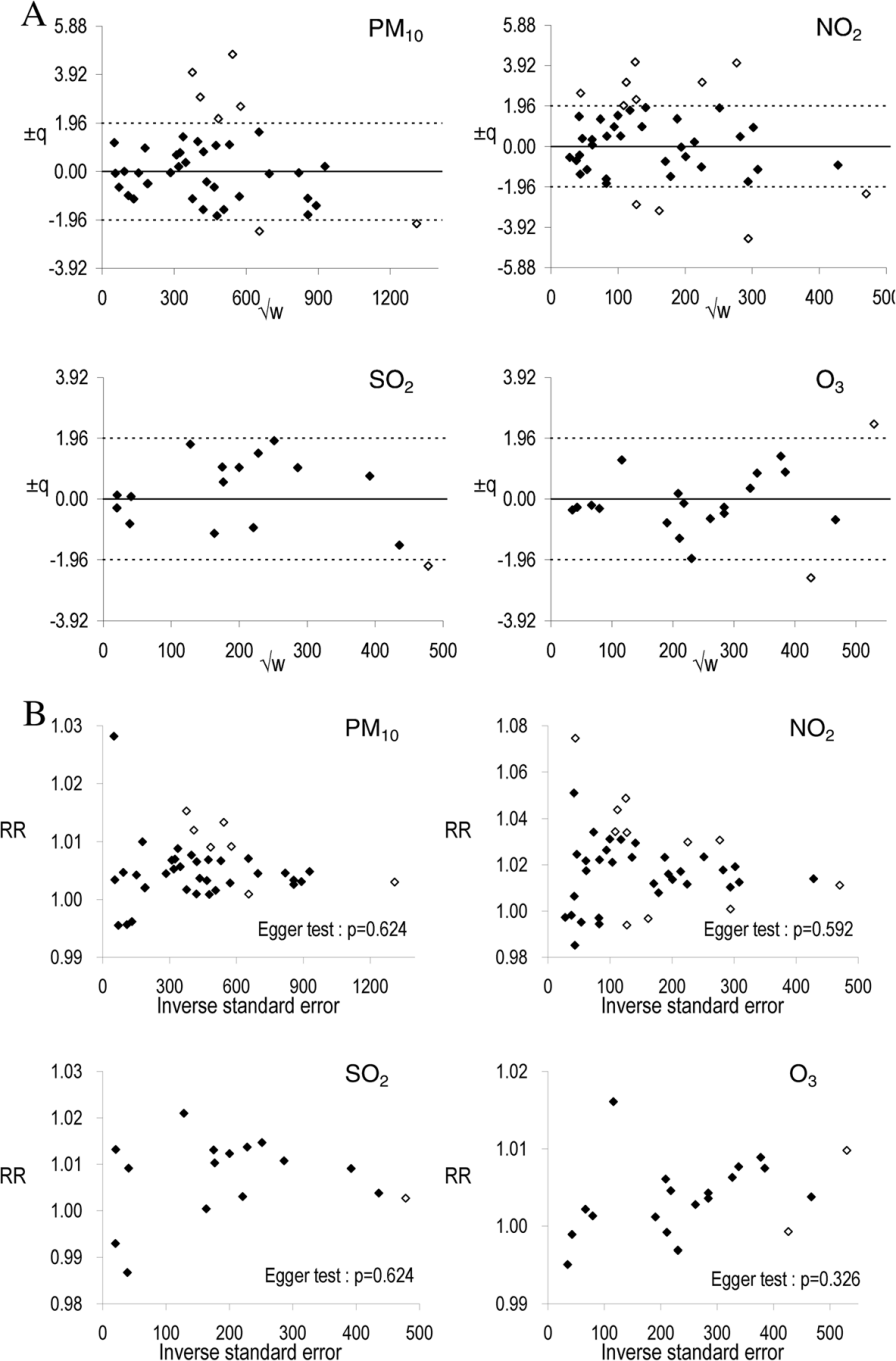
**Detection of heterogeneity and publication bias. A**. Influence plots, **B**. Funnel plots. ±q is the square root of heterogeneity measure of Q^2^ statistics [22]; √w is the square root of weights that based on estimate precisions; solid and hollow dots represent studies with ± q < −1.96 and ≥1.96 respectively.

The large amount of overall heterogeneity was due to studies in Beijing (±q = 4.01 and 4.74), Guangzhou (±q = 2.63 and 3.00), Hong Kong (±q = −2.11), Taiyuan (±q = −2.42) and Tianjin (±q = 2.14) for PM_10_; Beijing (±q = −3.12 and −4.47), Guangzhou (±q = 3.11), Hangzhou (±q = −2.82), Shanghai (±q = −2.29), Taiyuan (±q = 2.57 and 3.11), Tianjin (±q = 2.27), Wuhan (±q = 4.05 and 4.09) and Zhongshan (±q = 1.98) for NO_2_; Tianjin (±q = −2.16) for SO_2_; and Guangzhou (±q = 2.42) and Wuhan (±q = −2.53) for O_3_ (Figure 3A).

The number of studies in individual rubric was not sufficient for detection of publication bias. The heterogeneity in the meta-analysis was large in magnitude (*I*^*2*^ = 61% for PM_10_, 72% for NO_2_, 33% for SO_2_ and 23% for O_3_). Publication bias for all the pooled estimates was not significant for PM_10_ (Egger test: p = 0.624), NO_2_ (p = 0.592), SO_2_ (p = 0.624) and O_3_ (p = 0.326) (Figure 3B).

### Meta-regression

**Figure 4:**
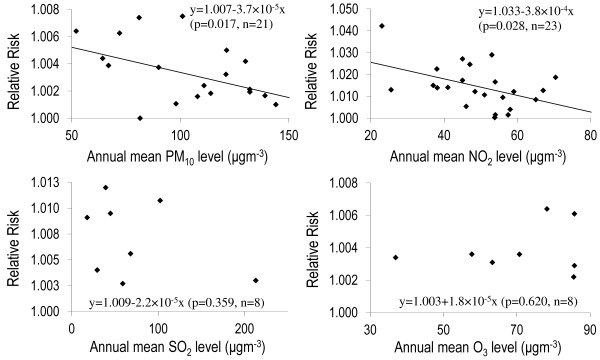
Meta-regression analysis of annual mean pollutant concentration and the relative risks (>1) of mortality for all natural causes in different studies.

Meta-regression of annual concentrations and the reported RR above the unity (>1) indicated inverse linear associations for PM_10_ (n = 21, β = −3.659 × 10^-5^, p = 0.017) and NO_2_ (n = 23, β = −3.79 × 10^-4^, p = 0.028) but no associations for SO_2_ (p = 0.359) and O_3_ (p = 0.620) (Figure 4).

## Discussion

To summarize, the reported RR that were statistically significant (p < 0.05) for mortality ranged from 1.0031 (all-cause associated with PM_10_) to 1.0140 (RD associated with NO_2_); for hospital admissions ranged from 1.0021 (CD associated with PM_10_) to 1.0162 (asthma associated with O_3_), and for birth outcomes from 1.0051 (stillbirth associated with O_3_) to 1.1189 (preterm-birth associated with SO_2_).

Most of the studies in Chinese populations under review revealed that the pollutants levels were higher than the WHO annual Air Quality Guidelines (AQG) [2] or the derived annual mean according to WHO short-term AQG [24]. Pooled RR from these studies are useful for health impact assessment because they were based on clinical records of physician-diagnosed diseases, birth outcomes and deaths, which were usually more reliable indicators of population health than measurements of physiological changes and other subtle health effects [66].

In the present review, our pooled RR were somehow consistent with the literature. The pooled RR of all causes mortality in all age groups for PM_10_, NO_2_, SO_2_ and O_3_ were differed from −5% to +68% of those recently reported in a meta-analysis on Asian countries by the *Health Effects Institute* (HEI). The pooled RR of CD and RD mortality were differed from −10% to +42% and −13% to +59% respectively [13,26,27,69]. The main difference from the HEI meta-analysis is that we included literatures that published in Chinese language and confined only to Chinese populations.

In total, we reviewed 23 different rubrics of adverse health outcomes based on population studies. The patterns of pooled RR of mortality and hospital admission for various rubrics were quite consistent with the order that NO_2_ > SO_2_ > PM_10_ > O_3_ per unit increase in concentration of each pollutant. Such order was found in the HEI report [13,67]. However, for all-cause mortality, CD and COPD, the RR of O_3_ was slightly higher than that of PM_10_; the RR of hospital asthma admissions was the highest for O_3_. For emergency room visits and admissions, the number of studies was insufficient to observe the pattern. The values of RR of mortality were also consistent for all pollutants in the order for RD > CD > all-causes, except for O_3_, and this pattern was also found elsewhere [13,67], supporting that health effects of air pollution in China are comparable to those shown in other parts of the world [67].

The range of RR (p < 0.05) of each rubric for the four pollutants (PM_10_, NO_2_, SO_2_ and O_3_) can be classified into three levels: (i) RR ranged from less than 1 to 2% including all causes, CD, RD (for hospital admissions and emergency room visits), cardiopulmonary diseases, cardiac diseases, cerebrovascular diseases, IHD (for hospitalization), heart failure (for emergency room visits), cardiac arrhythmias, COPD, asthma, acute RD, non-cardiopulmonary-non-accidental, diabetes, pneumonia, cancer, stillbirth and low birth weight. (ii) Rubrics with RR ranging from 1 to 4% included RD (for mortality and emergency admissions), IHD (for mortality), acute upper respiratory infections, acute pharyngitis, and influenza and pneumonia. (iii) Rubrics with RR ranging from 4 to 12% included heart failure (emergency admissions), hypertension and preterm birth. These ranges may provide a useful summary in specifying the health impacts due to every 10 μg/m^3^ increase in air pollution in Chinese populations.

Seasonality modified the effects of the pollutants, in that largest RR occurred during the winter season [68]. These findings in Chinese cities are similar to some other countries [69,70]. But different results may also be found in other countries such as the US and Netherlands [71,72], suggesting that an inadequate adjustment for confounding due to seasonality which varied from studies to studies may lead to variance in the effect estimates.

In meta-regression analysis we found some evidence of an inverse linear relationship between RR of mortality and the annual PM_10_ and NO_2_ concentration. This pattern indicates that the concentration-response relationship as a downward leveling off pattern rather than linear [73]. Similar pattern of inverse association has been observed in a cross-sectional study of lung function and exposure to indoor PM_2.5_ concentration [11]. This inverse relationship could be related to a saturation mechanism occurring at lower exposure levels [74] where both irreversible and reversible processes may simultaneously exist. This has been hypothesized that, as indicated by structural changes in airways [75,76], air pollutant that penetrate to the deepest part of the lung and cause alveolar epithelial injury are associated with both acute reversible inflammatory responses and cumulative irreversible pulmonary damage [11]. Explanation by the saturation hypothesis for cardiovascular diseases is also warranted [73]. Our findings suggest there are needs for further study to assess the inverse relationships using literatures in the world. We did not find any patterns yet for SO_2_ and O_3_ probably due to insufficient data for meta-regressions.

In the present review, there were sufficient Chinese studies on all-cause and cause-specific mortality for all pollutants to perform the meta-analysis; but studies on hospitalizations and emergency room visits as well as adverse birth outcomes and long-term effects were insufficient. The changes in levels of exposure and city background characteristics such as urbanization level, medical and hygiene standards have now been underway during the rapid economic development in the past decade. This may be a confounding factor that explains why at present the magnitude of the reported RR of long-term effects in China [63-65] is incomparable to those in the western countries. The evidence of long-term effects is therefore immature for drawing a definite conclusion. However, the aforementioned confounding factor is not applicable to the assessment of short-term effects, and so the reported RR of common disease rubrics in China, Taiwan and Hong Kong were largely similar and comparable to international findings [13,67].

The limitations of the present study may include search bias because we based our literature search on PubMed only, despite the fact that PubMed is a well known biomedical literature database and missing of important epidemiologic studies are less likely happened. Publication bias is also a common limitation for meta-analysis, in which “positive” results are more likely to be published than those the “negative” results, leads to the censoring of studies with non-significant results [77]. However, in our study publication bias was not observed. We detected some heterogeneity due to several studies for each pollutant. The potential reasons may include the difference in age structure in different populations, study designs and statistical methods for analysis. For differences in ICD codes, we identified that some studies used quite different codes or reported no codes [28,29,43,49,78,79] in defining the health outcome, making them almost incomparable with the majority of other studies. When excluded studies using different codes, the pooled RR have changed only about 1-7%. Hence, we based our interpretations on the pooled RR using the all the ICD codes.

## Conclusion

Ambient air pollution in Chinese populations is poor when compared with well developed countries in Europe, North America, and Asia-Pacific region. For short-term exposure, the pooled relative risks estimates of the major health effects including all causes, cardiovascular and respiratory diseases were comparable with findings from studies worldwide. Inverse linear associations between short-term relative risks of mortality and annual mean PM_10_ and NO_2_ concentrations suggests that the concentration response relationship may be non-linear and characterized by a concave downward curve leveling off at high concentration. These are useful for developing health evidence-based risk communications and health impact assessments. However, evidence on long-term effects and adverse birth outcomes are still insufficient for meta-analysis and decision-making in environmental policy and management.

## Competing interests

The author(s) declare that they have no competing interests.

## Authors’ contributions

HKL initiated the study and designed the analytical methodology. HT collected the data and conducted the data analysis with HKL. HT and HKL drafted the manuscript. CMW provided critical comments and edited on the draft. All authors read and approved the final manuscript.

## Pre-publication history

The pre-publication history for this paper can be accessed here:

http://www.biomedcentral.com/1471-2458/13/360/prepub

## Supplementary Material

Additional file 1 **(1) Forest plot for the all-causes mortality in different studies; (2) Analysis of differences in ICD used in the literatures; (3) 60 locations without relevant literatures; (4) Older literatures excluded from the pooled relative risks of meta-analysis in Table **1. Click here for file
