# Supplementary material for: Meta-analysis of adverse health effects due to air pollution in Chinese populations
Source: BMC Public Health. 2013 Apr 18;13:360. doi: 10.1186/1471-2458-13-360 (PMC3698155; doi:10.1186/1471-2458-13-360)
Supplement: Additional file 1 — (1) Forest plot for the all-causes mortality in different studies; (2) Analysis of differences in ICD used in the literatures; (3) 60 locations without relevant literatures; (4) Older literatures excluded from the pooled relative risks of meta-analysis in Table 1. [file 1471-2458-13-360-S1.docx]

**Forest plot for the all-causes mortality in different studies**


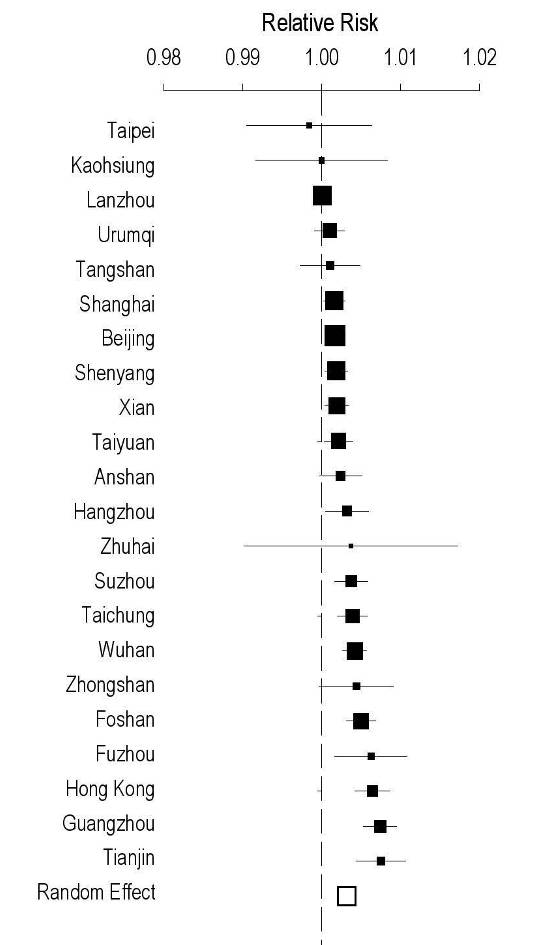

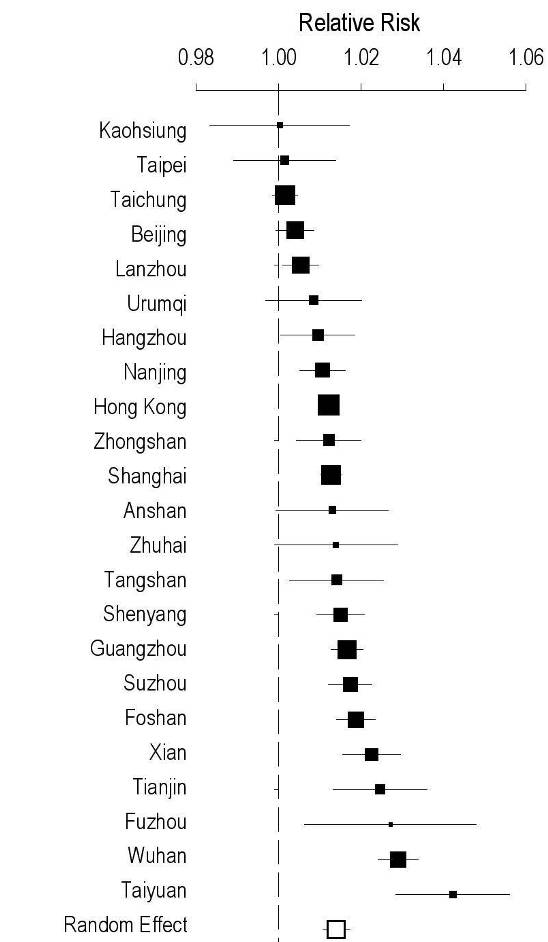


NO_2_

PM_10_


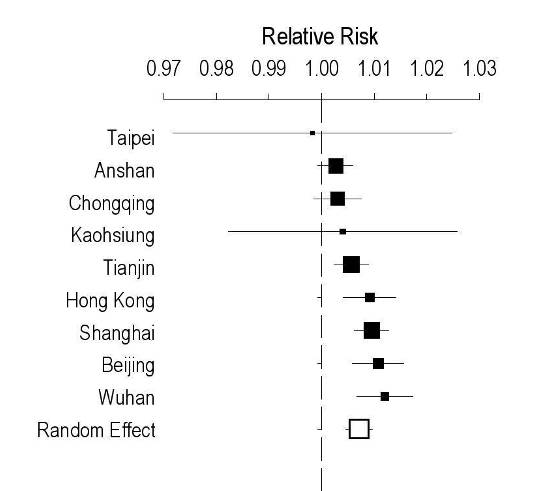

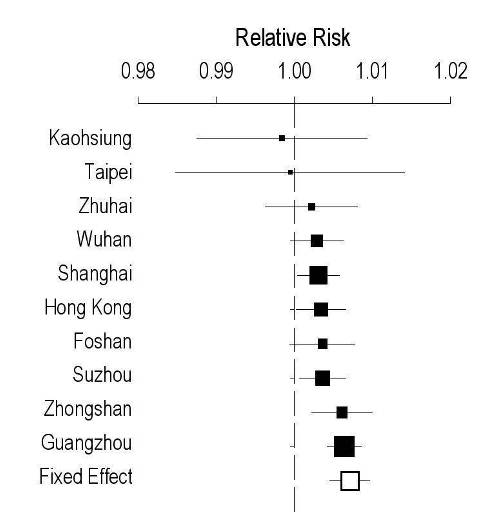


O_3_

SO_2_

**Analysis of differences in ICD used in the literatures**

| Health effects | ICD-9 | ICD-10 | Ref # | Difference(%) |
| --- | --- | --- | --- | --- |
| All-causes | 1-799 | A00-R99 | [[33](#_ENREF_33), [34](#_ENREF_34), [45](#_ENREF_45), [46](#_ENREF_46), [50](#_ENREF_50), [52-65](#_ENREF_52), [67](#_ENREF_67)] | 0 |
|  | *1-999* | A00-T98 | [[71](#_ENREF_71)] | 25.0 |
|  |  |  |  |  |
| Cardiovascular diseases | 390-459 | I00-I99 | [[33](#_ENREF_33), [34](#_ENREF_34), [50](#_ENREF_50), [52-54](#_ENREF_52), [56-59](#_ENREF_56), [61](#_ENREF_61), [67](#_ENREF_67), [68](#_ENREF_68), [72-74](#_ENREF_72)] | 0 |
|  | 390-414,417-448 | *I00-I25,I27-I78* | [[45](#_ENREF_45), [46](#_ENREF_46), [60](#_ENREF_60)] | 18.6 |
|  | 410-444 | *I20-I74* | [[76](#_ENREF_76)] | 50.0 |
|  | 410-411,414,430-437 | *I21-I25,I60-I68* | [[62](#_ENREF_62)] | 84.3 |
|  |  | |  |  |
| Ischemia heart diseases | 410-414 | I20-I25 | [[54](#_ENREF_54), [58](#_ENREF_58), [72](#_ENREF_72), [73](#_ENREF_73)] | 0 |
|  | 410,411,414 | *I21-I25* | [[48](#_ENREF_48)] | 40.0 |
| Stroke/ Cerebrovascular diseases | 430-438 | I60-I69 | [[53](#_ENREF_53), [54](#_ENREF_54), [58](#_ENREF_58), [61](#_ENREF_61), [65](#_ENREF_65), [72](#_ENREF_72)] | 0 |
|  | 430-437 | *I60-I68* | [[48](#_ENREF_48)] | 11.1 |
| Hypertension | *401* | I10 | [[80](#_ENREF_80), [81](#_ENREF_81)] | NA |
| Cardiac diseases | 390-398,410-429 | I00-I09,I20-I52 | [[53](#_ENREF_53), [54](#_ENREF_54), [58](#_ENREF_58), [65](#_ENREF_65)] | 0 |
|  | 390-429 | *I00-I52* | [[73](#_ENREF_73)] | 37.9 |
| Heart failure | 428 | I50 | [[48](#_ENREF_48), [73](#_ENREF_73)] | NA |
| Cardiac arrhythmias | *427* | I47-I49 | [[73](#_ENREF_73)] | NA |
|  |  |  |  |  |
| Respiratory diseases | 460-519 | J00-J98/99 | [[33](#_ENREF_33), [34](#_ENREF_34), [52-58](#_ENREF_52), [62-66](#_ENREF_62), [68](#_ENREF_68), [71](#_ENREF_71), [73](#_ENREF_73), [78](#_ENREF_78), [79](#_ENREF_79)] | 0 |
|  | 460-466,470-478,480-487,490-496 | *J00-06,J09-J18, J20-J21,J30-J47* | [[48](#_ENREF_48)] | 50.0 |
| Asthma | 493 | J45-J46 | [[54](#_ENREF_54), [74](#_ENREF_74)] | NA |
| Chronic obstructive pulmonary diseases | 490-496 | J40-J47 | [[53](#_ENREF_53), [54](#_ENREF_54), [69](#_ENREF_69)] | 0 |
|  | 490-493 | *J40-J45* | [[45](#_ENREF_45), [46](#_ENREF_46)] | 42.9 |
|  | 491,492,496 | *J41-J43,J47* | [[74](#_ENREF_74)] | 57.1 |
| Influenza and pneumonia | 480-487 | *J09-J18* | [[54](#_ENREF_54), [58](#_ENREF_58)] | 0 |
|  | 466,480-487 | *J10-J22* | [[72](#_ENREF_72)] | 12.5 |
| Pneumonia | 486 | *J18* | [[72](#_ENREF_72)] | NA |
| Acute upper respiratory infections | *460-466* | J00-J06 | [[78](#_ENREF_78)] | NA |
| Acute respiratory diseases | 460-466,480-487 | *J00-J06,J09-J18* | [[54](#_ENREF_54)] | NA |
| Acute pharyngitis | *462* | J02 | [[78](#_ENREF_78)] | NA |
|  |  |  |  |  |
| Cardiopulmonary disease | 390-519 | I00-J98 | [[50](#_ENREF_50), [58](#_ENREF_58), [65](#_ENREF_65)] | NA |
| Diabetes | 250 | *E10-E14* | [[70](#_ENREF_70)] | NA |
| Cancer | 140-208 | *C00-C96* | [[45](#_ENREF_45), [46](#_ENREF_46), [50](#_ENREF_50)] | NA |
| Non-cardiopulmonary, natural | 1-389,520-799 | A00-H95,K00-R99 | [[54](#_ENREF_54), [58](#_ENREF_58), [65](#_ENREF_65)] | NA |
| Accidental | 800-999 | S00-T98 | [[54](#_ENREF_54), [58](#_ENREF_58)] | NA |
| Stillbirths | 740-758 | *Q00-Q99* | [[26](#_ENREF_26)] | NA |
|  |  |  |  |  |

*Note.* Difference (%) in ICD-9 was shown. ICD codes in *Italic* were translated from the adjacent non-italic codes based on two reference sources (<http://apps.who.int/classifications/icd10/browse/2010/en> & [http://icd9cm.chrisendres.com](http://icd9cm.chrisendres.com/)). NA, Not applicable.

**60 locations without relevant literatures:**

Anqing, Beihai, Bengbu, Changchun, Changde, Changsha, Changzhou, Chengdu, Dali, Dalian, Dandong, Fuzhou, Ganzhou, Guilin, Guiyang, Haikou, Harbin, Hefei, Hohhot, Hsinchu, Huizhou, Jilin, Jinan, Jinhua, Jining, Jinzhou, Jiujiang, Kunming, Luoyang, Luzhou, Macau, Mudanjiang Nanchang, Nanchong, Nanjing, Nanning, Ningbo, Pingdingshan, Qinhuangdao, Sanya, Shaoguan, Shijiazhuang, Tainan, Tangshan, Taoyuan, Ürümqi, Wenzhou, Wuxi, Xiamen, Xiangyang, Xining, Xi'an, Xuzhou, Yangzhou, Yantai, Yichang, Yinchuan, Yueyang, Zhanjiang and Zunyi.

**Additional table: Relative risks of all-cause mortality in all age groups due to air pollution in older period of the same city. They were excluded from Table 1 and not pooled in the meta-analysis.**

| **Ref#** | **City** | **Period** | **PM_10_** |  | **NO_2_** |  | **SO_2_** |  | **O_3_** |  |
| --- | --- | --- | --- | --- | --- | --- | --- | --- | --- | --- |
|  |  |  | **RR** | **SE** | **RR** | **SE** | **RR** | **SE** | **RR** | **SE** |
| [1,2] | Anshan | 2001-2004 | 1.0007 | 12 | 1.0111 | 57 |  |  |  |  |
| [1,2] | Guangzhou | 2007-2008 | 1.0115* | 12 | 1.0204* | 19 |  |  |  |  |
| [3] | Hong Kong | 1995-1997 | 1.0037* | 19 | 1.0091* | 12 | 1.0111* | 38 | 1.0018 | 18 |
| [1,2] | Shanghai | 2001-2004 | 1.0030* | 5 |  |  |  |  |  |  |
| [4] |  | 2002-2003 | 1.0053* | 16 |  |  |  |  |  |  |
| [5] |  | 2000-2001 | 1.0030* | 10 | 1.0200* | 36 | 1.0160* | 26 |  |  |
| [6] | Taiyuan | 2004-2005 | 1.0025* | 11 |  |  |  |  |  |  |
| [7,8] | Tianjin | 2005-2007 | 1.0045* | 12 | 1.0094* | 39 |  |  |  |  |
| [9] | Wuhan | 2000-2004 | 1.0043* | 10 | 1.0196* | 34 |  |  |  |  |

*Note. RR*, Relative risk per 10 μg/m^3^ increase in pollutant concentration; *SE*, Standard error in 10^-4^. * indicated statistically significant at alpha = 0.05. 95%Cl = 1.96*SE ± RR.

**References:**

1. Chen R, Kan H, Chen B, Huang W, Bai Z, Song G, Pan G, Group oBotCC: **Association of particulate air pollution with daily mortality: the china air pollution and health effects study.** *Am J Epidemiol* 2012, **175**:1173–1181.
2. Chen R, Samoli E, Wong CM, Huang W, Wang Z, Chen B, Kan H, CCG: **Associations between short-term exposure to nitrogen dioxide and mortality in 17 chinese cities: the china Air pollution and health effects study (CAPES).** *Environ Int* 2012, **45C**:32–38.
3. Wong CM, Ma S, Hedley AJ, Lam TH: **Effect of air pollution on daily mortality in Hong Kong.** *Environ Health Perspect* 2001, **109**:335–340.
4. Wong CM, Thach TQ, Chau PY, Chan EK, Chung RY, Ou CQ, Yang L, Peiris JS, Thomas GN, Lam TH, *et al*: **Part 4. Interaction between air pollution and respiratory viruses: time-series study of daily mortality and hospital admissions in Hong Kong.** *Res Rep Health Eff Inst* 2010:283–362.
5. Kan H, Chen B: **Air pollution and daily mortality in Shanghai: a time-series study.** *Arch Environ Health* 2003, **58**:360–367.
6. Zhang YP, Zhang ZQ, Liu XH, Zhang XP, Feng BQ, Li HP: **Concentration-response relationship between particulate air pollution and daily mortality in Taiyuan.** *Beijing Da Xue Xue Bao* 2007, **39**:153–157.
7. Zhang YS, Zhou MG, Jia YP, Hu YS, Zhang JL, Jiang GH, Pan XC: **Time-series analysis of association between inhalable particulate matter and daily mortality among urban residents in Tianjin.** *Zhonghua Liu Xing Bing Xue Za Zhi* 2010, **31**:544–548.
8. Zhang YS, Zhou MG, Jia YP, Hu YS, Zhang JL, Jiang GH, Pan XC: **Time-series analysis on the association between gaseous air pollutants and daily mortality in urban residents in Tianjin.** *Zhonghua Liu Xing Bing Xue Za Zhi* 2010, **31**:1158–1162.
9. Qian Z, He Q, Lin HM, Kong L, Zhou D, Liang S, Zhu Z, Liao D, Liu W, Bentley CM, *et al*: **Part 2. Association of daily mortality with ambient air pollution, and effect modification by extremely high temperature in Wuhan, China.** *Res Rep Health Eff Inst* 2010:91–217.
